# Developmental exposure to a mixture of perfluoroalkyl acids (PFAAs) affects the thyroid hormone system and the bursa of Fabricius in the chicken

**DOI:** 10.1038/s41598-019-56200-9

**Published:** 2019-12-24

**Authors:** Anna Mattsson, Sofia Sjöberg, Anna Kärrman, Björn Brunström

**Affiliations:** 1 Department of Environmental Toxicology, Uppsala University, Uppsala, Sweden; 2 School of Science and Technology, Örebro University, Örebro, Sweden

**Keywords:** Environmental sciences, Environmental impact

## Abstract

Perfluoroalkyl acids (PFAAs) are ubiquitous environmental contaminants and eggs and nestlings of raptors and fish-eating birds often contain high levels of PFAAs. We studied developmental effects of a mixture of ten PFAAs by exposing chicken embryos to 0.5 or 3 μg/g egg of each compound in the mixture. Histological changes of the thyroid gland were noted at both doses and increased expression of mRNA coding for type III deiodinase was found at 0.5 μg/g egg. Serum concentrations of the free fraction of thyroid hormones (T3 and T4) were reduced by the PFAA mixture at 3 µg/g egg, which is in line with a decreased synthesis and increased turnover of thyroid hormones as indicated by our histological findings and the decreased mRNA expression of type III deiodinase. The relative weight of the bursa of Fabricius increased at a dose of 3 μg/g egg in females. The bursa is the site of B-cell development in birds and is crucial for the avian adaptive immune system. Analysis of plasma and liver concentrations of the mixture components showed differences depending on chain length and functional group. Our results highlight the vulnerability of the thyroid hormone and immune systems to PFAAs.

## Introduction

The perfluoroalkyl acids (PFAAs) comprise a large group of synthetic environmental contaminants that raise great concern regarding possible adverse health effects in humans and wildlife. PFAAs is the class of per- and polyfluoroalkyl substances (PFAS) most commonly found in biota. These substances have been produced for more than six decades and are extensively used as components in many different applications and consumer products, for instance in firefighting foams, in cosmetics, as surfactants, and as surface protecting agents for e.g. textiles and food packaging^1^.

A PFAA consists of a fluorinated alkyl chain of varying length with a hydrophilic group, commonly a carboxyl or a sulfonyl hydroxide group. Due to the strong carbon-fluorine bonds, these compounds are exceptionally stable and persistent in the environment^2^. PFAAs are divided into short- and long-chain PFAAs; the long-chain PFAAs are defined as perfluoroalkyl carboxylic acids with ≥7 perfluorinated carbons (≥8 carbons in total) and perfluoroalkane sulfonic acids with ≥6 perfluorinated carbons (≥6 carbons in total)^3^. The long-chain PFAAs show greater potential for accumulation in wildlife and humans than the short-chain PFAAs^4–8^. Because of these properties, combined with their extensive use, the long-chain PFAAs are now found globally in biota, including humans^9–11^.

The most common and well-studied PFAAs are perfluorooctanesulfonic acid (PFOS) and perfluorooctanoic acid (PFOA). Due to concerns regarding potential health and environmental effects of PFOS and PFOA there have been regulatory and voluntary actions to phase them out. For instance, PFOS is since 2009 regulated globally under the Stockholm convention on Persistent Organic Pollutants (POPs), and PFOA and perfluorohexanesulfonic acid (PFHxS) are currently under review for listing. However, levels of these PFAAs are still high and some of the other long-chain PFAAs continue to show increasing concentrations in humans and wildlife^12–14^. High levels of PFOS and other long-chain PFAAs are found globally in raptors and fish-eating birds^15^. In Sweden, ng/g to µg/g levels have been reported in bird eggs and in plasma from hatchlings^16–19^. Long-chain PFAAs are readily transferred from the mother to the developing embryo via egg deposition^16,20,21^. In mammals, PFAAs are transferred from the mother to the offspring via placenta^22,23^ and breast milk^24,25^.

PFOS and PFOA have in animal experiments and epidemiological studies been associated with a range of adverse effects, including hepatotoxicity, developmental perturbations, altered lipid metabolism, thyroid hormone disruption, and immunotoxicity, as reviewed by Borg and Håkansson^26^. A few PFAAs have been studied in avian models and there are indications that some of them, e.g. PFOA, PFOS and PFHxS, induce various developmental effects including cardiotoxicity, altered fatty acid metabolism, thyroid hormone disruption and immunotoxicity^27–30^.

Because of the vital importance of the thyroid hormone and immune systems throughout life, impairment of the functions of these by environmental chemicals may result in detrimental effects in birds and other vertebrates. In the present study, we used the chicken embryo as a model to further explore potential developmental effects of PFAAs on the thyroid hormone and immune systems in birds. Embryos were exposed *in ovo* to a PFAA mixture consisting of eight perfluoroalkyl carboxylic acids and two perfluoroalkyl sulfonic acids that have been found in human fetal organs^22^, in serum from infants^24^ and in eggs or embryo tissues of wild animals, including birds^9,16,17,20,31^. All components of the mixture are long-chain PFAAs according to the definition by Buck *et al*., except for perfluorohexanoic acid (PFHxA) which is defined as a short-chain PFAA^3^. We found that embryonic exposure to the PFAA mixture affected thyroid hormone functions and caused a sex-dependent change in the weight of the immune organ bursa of Fabricius. The distribution of the components of the injected mixture to embryo liver and plasma was dependent on chain length and hydrophilic group.

## Results

### Survival and weights of body, liver and spleen

Embryos were exposed by air sac injection on embryonic day four (E4) and were euthanized and dissected on E18. Mortality was low in all groups; 2/21 (10%) in the control, 1/21 (5%) in the group exposed to 0.5 µg PFAA/g egg and 1/20 (5%) in the group exposed to 3 µg PFAA/g egg. Body weight, liver-somatic index and spleen-somatic index were not affected by treatment (Table 1).

**Table 1:** Body weight and relative organ weights on embryonic day 18.

Group	No of embryos	Body weight (g)	Liver-somatic index (%)	Spleen-somatic index (%)
Controls	19	24.9 ± 1.9	2.3 ± 0.2	9.6 ± 1.9
PFAA (0.5 µg/g egg)	20	25.5 ± 1.5	2.3 ± 0.1	10.3 ± 1.6
PFAA (3.0 µg/g egg)	19	24.4 ± 1.5	2.3 ± 0.1	9.2 ± 1.7

Data presented as mean ± standard deviation.

### Liver histology

Histological sections of livers were examined visually by microscopy. Pathological changes, such as fatty change, disorganisation of tissue structure, necrosis/apoptosis, or invasion by leucocytes were not found in any of the groups.

### Thyroid gland morphology

**Figure 1:**
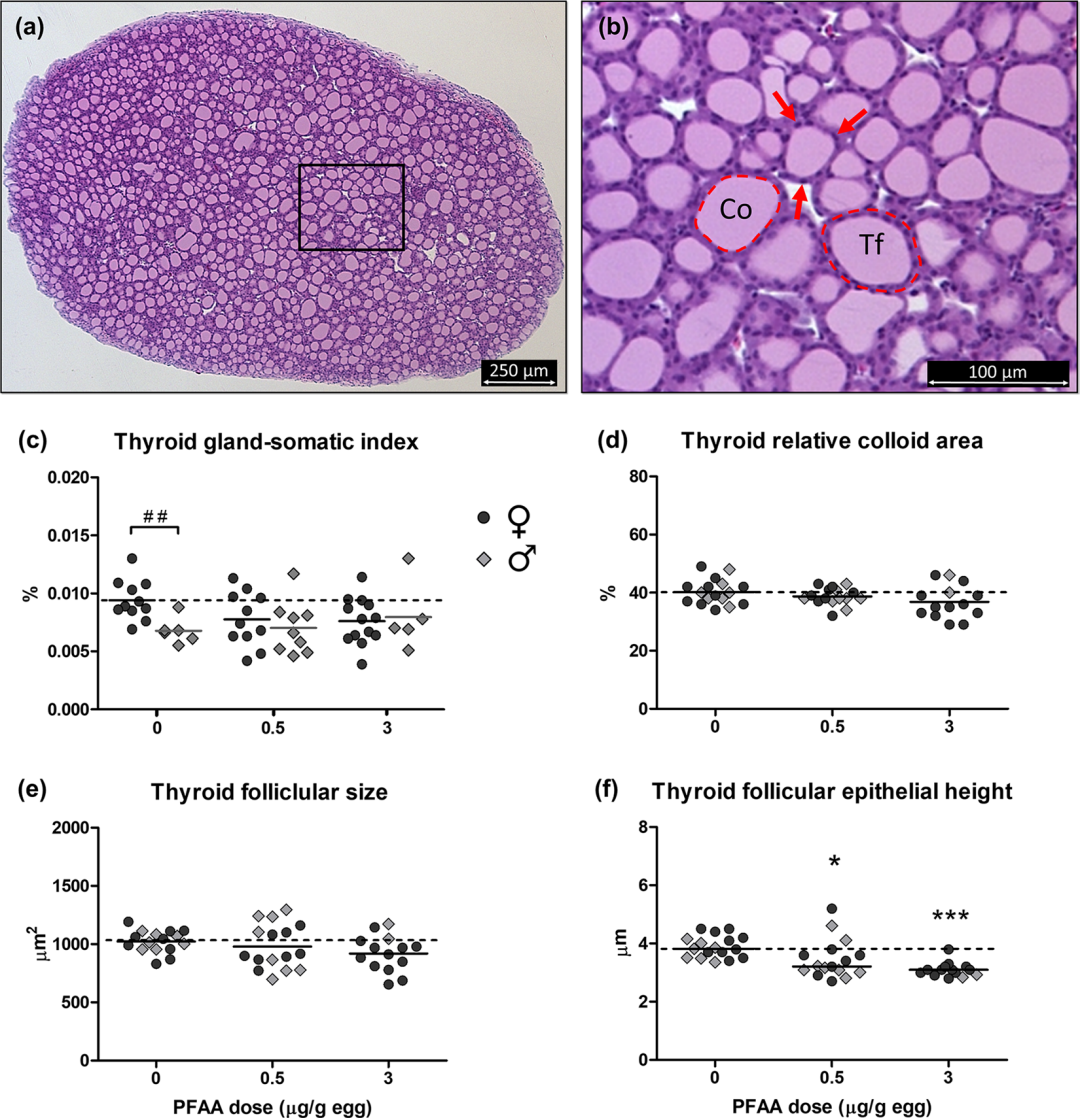
Thyroid gland morphology. (**a**) Histological section of the thyroid gland from an 18-day-old control chicken embryo taken along the longitudinal axis of the organ. Stain: haematoxylin and eosin. The thyroid gland consists mainly of thyroid follicles surrounded by sparse amount of connective tissue. **(b)** Magnified view of the area within the rectangle in (**a**). The thyroid follicle (Tf) consists of epithelial cells (indicated by arrows) and a lumen filled with colloid (Co). The thyroid hormones thyroxine and triiodothyronine (T4 and T3) are produced by the epithelial cells and are stored in the follicular lumen in the form of the precursor thyroglobulin as part of the colloid. **(c)** Thyroid gland-somatic index, **(d)** thyroid relative colloid area, **(e)** thyroid follicular size, and **(f)** thyroid follicular epithelial height in chicken embryos exposed *in ovo* to the vehicle (DMSO) or a mixture of PFAAs at 0.5 or 3 µg/g egg. Symbols represent individual embryos. Solid lines show group mean for females (♀) and males (♂) separately in (**c**), mean of both sexes in (**d**,**e**) and median of both sexes in (**f**). The dashed line corresponds to the control mean/median of females in (**c**) and of both sexes in (**d**–**f**). ^##^Sex difference (t-test, p < 0.01). *Treatment effect (Dunnett’s post-test, p < 0.05). ***Treatment effect (Dunnett’s post-test, p < 0.001). The post-test for linear trend showed a significant decrease with increasing dose for relative colloid area (both sexes, p < 0.05), follicular size (females, p < 0.05) and epithelial height (both sexes, p < 0.001).

In birds, the thyroid gland is a paired oval organ, which consists of connective tissue surrounding follicles producing and storing thyroid hormones (Fig. 1a,b). The hormones are stored in the form of colloid in the follicular lumen. The relative weight of the left thyroid gland, i.e. the thyroid gland-somatic index, showed no significant sex difference, treatment effect or interaction between sex and treatment (two-way ANOVA; Fig. 1c). However, the mean thyroid gland-somatic index was 28% lower in males than in females in the control group and this was a significant difference (t-test, p < 0.01).

The visual histological examination revealed no overt histopathological changes or any difference between treatment groups. The relative colloid area and the follicular size showed no sex difference and the PFAA-treated groups did not differ significantly from the control (Fig. 1d,e). However, the relative colloid area showed a significant linear trend of decrease with dose (p < 0.05). There was also a significant linear trend of decreased thyroid follicular size in females (p < 0.05), but not in males or both sexes combined. The follicular epithelial height was significantly reduced in the groups treated with the PFAA mixture compared with the epithelial height in the control group (Fig. 1f). The reduction was 11% at 0.5 µg/g egg (p < 0.05) and 20% at 3 µg/g egg (p < 0.001) and there was a significant linear trend of decrease with dose (p < 0.001).

### Thyroid hormone concentrations

**Figure 2:**
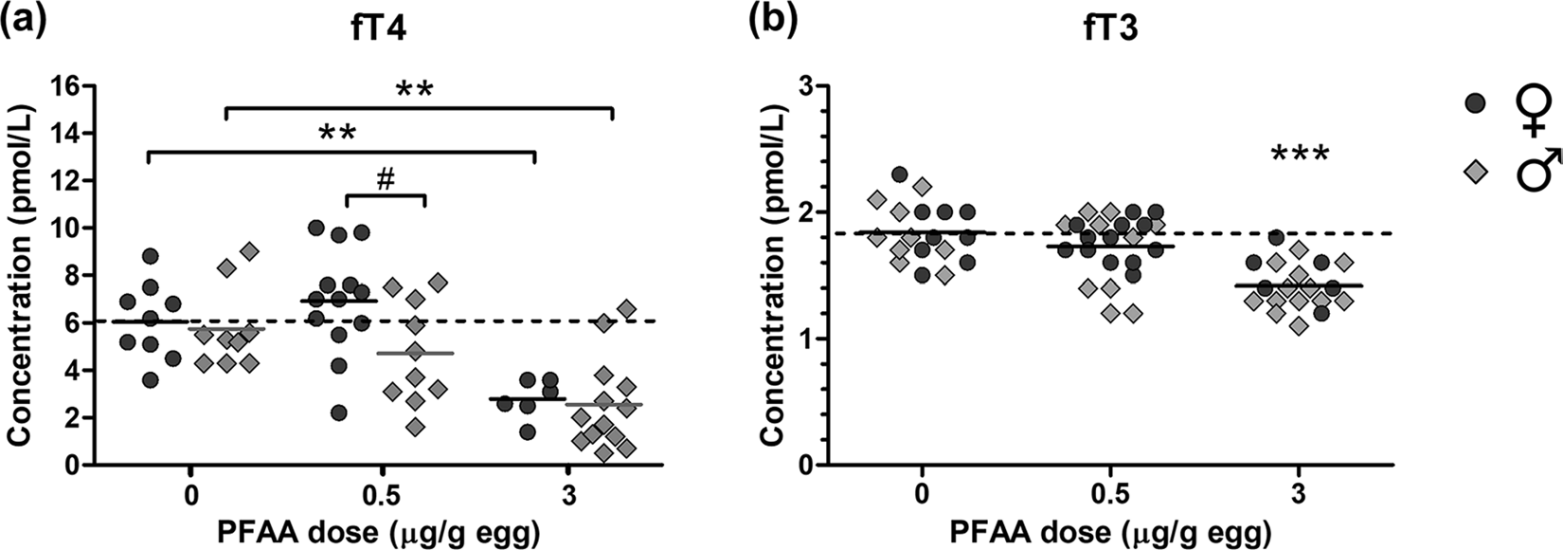
Serum concentrations of free T4 (**a**) and T3 (**b**) in female and male chicken embryos. Embryos were exposed *in ovo* to the vehicle (DMSO), or a mixture of PFAAs at 0.5 or 3 µg/g egg. Symbols represent individual embryos. Solid lines show group means for females (♀) and males (♂) separately in (**a**) and for both sexes combined in (**b**). The dashed line corresponds to the control mean of females in (**a**) and to the control mean of both sexes in (**b**). **Treatment effect (Dunnett’s post-test, p < 0.01). ***Treatment effect (Dunnett’s post-test, p < 0.001). #Sex difference (Bonferroni’s post-test, p < 0.05). The post-test for linear trend showed a significant decrease of fT4 in males (p < 0.001) and of fT3 in both sexes combined (p < 0.001) with increasing dose.

Serum concentrations of the free fraction of thyroxine and triiodothyronine (T4 and T3) were determined on E18 in a follow-up experiment (Fig. 2). The mean concentration of free T4 (fT4) was higher in females than in males in the group exposed to 0.5 µg/g egg (p < 0.05), but showed no sex differences in the other groups (Fig. 2a). Concentrations of fT4 were significantly reduced in both females and males exposed to PFAA at 3 µg/g egg compared to control (p < 0.01; Fig. 2a). The average fT4 concentration decreased from ~6 pmol/L in the control to ~2.7 pmol/L in the group exposed to 3 µg PFAA/g egg. In males, there was also a significant linear trend of a decrease of fT4 with exposure dose (p < 0.001).

There was no indication of sex differences in concentrations of free T3 (fT3), and males and females were therefore analysed together (Fig. 2b). The concentration of fT3 was significantly reduced by exposure to the PFAA mixture at the highest dose (p < 0.01). The mean concentration was 1.8 pmol/L in the control group, 1.7 pmol/L in the group exposed to 0.5 μg/g egg and 1.4 pmol/L in the group exposed to 3 μg/g egg. There was a significant linear trend of decrease of fT3 with dose (p < 0.001).

### Hepatic gene expression

**Figure 3:**
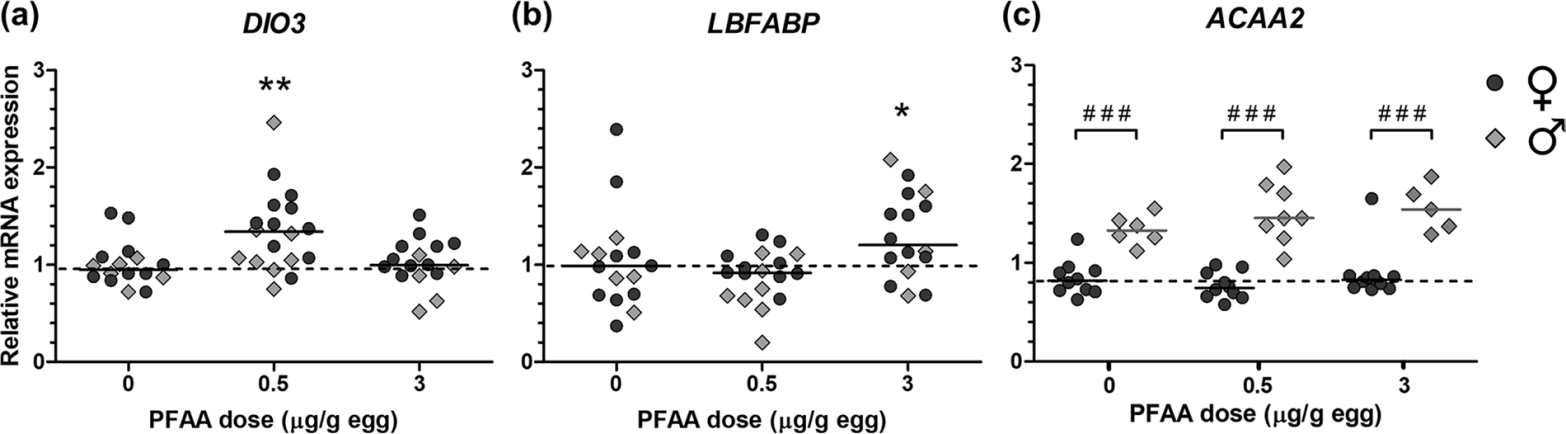
Relative mRNA expression of *DIO3, LBFABP* and *ACAA2* in chicken embryos. Embryos were exposed *in ovo* to the vehicle (DMSO), or a mixture of PFAAs at 0.5 or 3 µg/g egg. Symbols represent individual embryos. Solid lines show group medians for both sexes combined in (**a**,**b**) and for females (♀) and males (♂) separately in (**c**). The dashed line corresponds to the control median of both sexes in (**a**,**b**) and to the control median of females in (**c**). **Treatment effect (Dunnett’s post-test, p < 0.01). *Treatment effect (Dunn’s post-test, p < 0.05). ###Sex difference (Bonferroni’s post-test, p < 0.001).

Relative mRNA concentrations of *THRA* (thyroid hormone receptor, alpha)*, DIO1* (iodothyronine deiodinase 1)*, TTR* (transthyretin, transcript variants 1 and 2)*, LXR-A* (liver X receptor alpha) and *FABP5* (fatty acid-binding protein 5) did not differ between the sexes and they were not affected by treatment. The data is provided in the online Supplementary Table S3. Relative mRNA concentrations of *DIO3* (iodothyronine deiodinase 3)*, LBFABP* (liver basic fatty acid binding protein) and *ACAA2* (acetyl-CoA acyltransferase 2) are shown in Fig. 3. *DIO3* mRNA expression was significantly increased by the PFAA mixture at 0.5 µg/g egg (p < 0.01) but not at 3 µg/g egg (Fig. 3a). *LBFABP* mRNA expression was significantly increased by the PFAA mixture at 3 µg/g egg (p < 0.05) but not at 0.5 µg/g egg (Fig. 3b). *ACAA2* mRNA expression was markedly higher in males than in females in all groups (p < 0.001), but was not affected by PFAA treatment (Fig. 3c).

### Bursa of fabricius morphology

**Figure 4:**
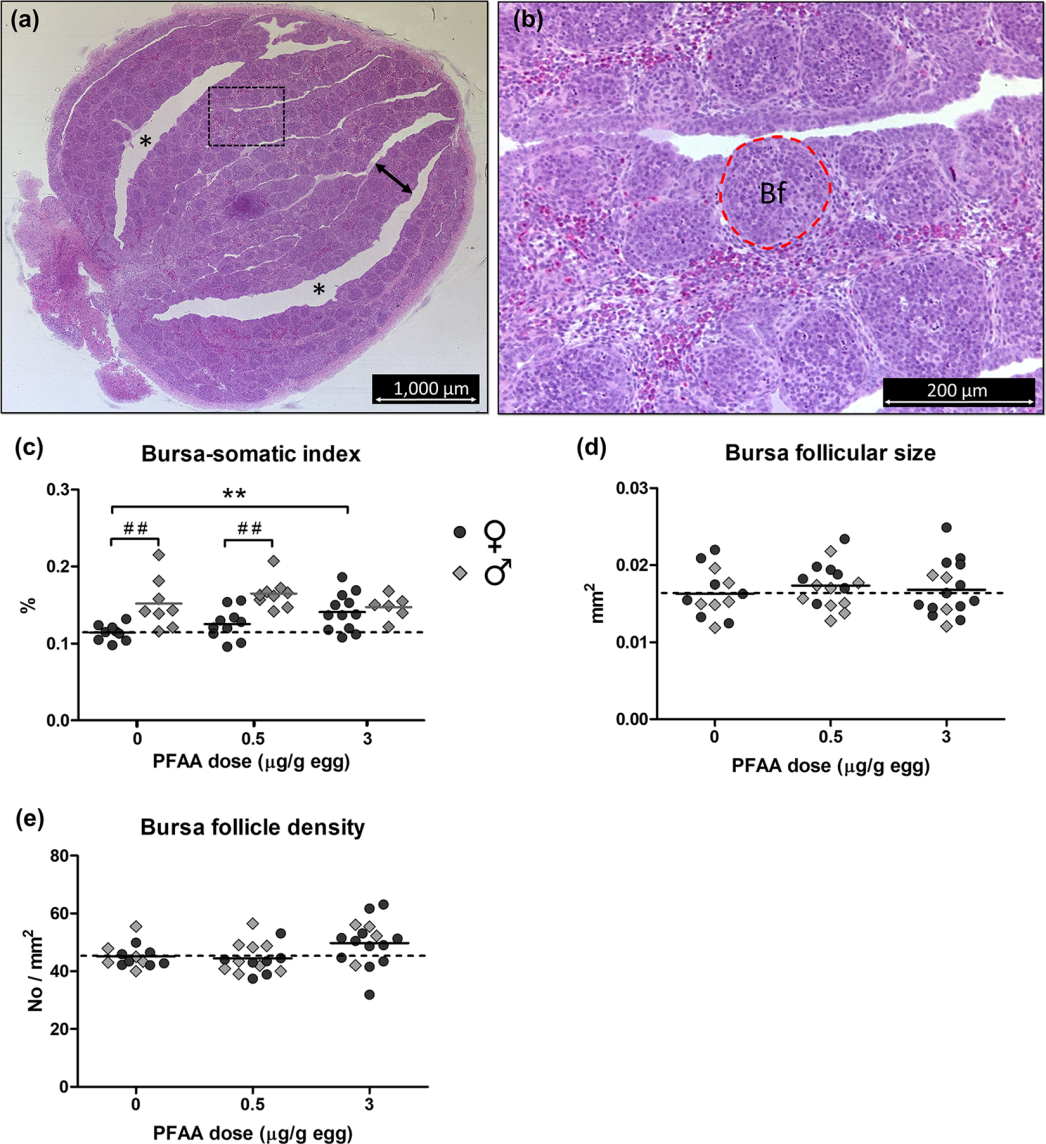
Bursa of Fabricius morphology. (**a**) Histological section of the bursa of Fabricius from an 18-day-old control chicken embryo taken along the longitudinal axis of the organ. Stain: haematoxylin and eosin. The bursa consists mainly of folds/plicae (indicated by arrow), which are separated by bursal lumen (indicated by *). **(b)** Magnified view of the area within the rectangle in (**a**) with one of the bursal follicles outlined (Bf). The bursal follicle is the site of B-lymphocyte development in birds. **(c)** Bursa-somatic index, **(d)** bursa follicular size, and **(e)** bursa follicle density in chicken embryos exposed *in ovo* to vehicle (DMSO), or a mixture of PFAAs at 0.5 or 3 µg/g egg. Symbols represent individual embryos. Solid lines show group mean for females (♀) and males (♂) separately in (**c**), and grand mean for each exposure group in (**d**,**e**). The dashed line corresponds to the control mean of females in (**c**), and of both sexes in (**d**,**e**). ^##^Sex difference (Bonferroni’s post-test, p < 0.01). **Treatment effect (Dunnett’s post test, p < 0.01). There was a significant trend of increased bursa-somatic index with increasing dose in females (post-test for linear trend, p < 0.01).

PFAA treatment did not induce any obvious histopathological changes in the bursa. The bursa contains folds of connective tissue, i.e. plicae, which at the examined age (E18) are filled with bursal follicles organized into two layers (Fig. 4a,b). There was no obvious difference between the treatment groups in shape of plicae or lumen area. The histomorphometric analyses showed that the size of individual follicles and follicle density were not significantly affected by treatment and did not differ between females and males (Fig. 4d,e). The relative weight of the bursa, i.e. the bursa-somatic index, was significantly lower in females than in males in both the control and in the group exposed to PFAA at 0.5 µg/g egg (p < 0.01; Fig. 4c). There was no such sex difference in the group exposed to PFAA at 3 µg/g egg. In females, the mean bursa-somatic index was 18% higher in the group exposed to 0.5 µg PFAA/g egg and 30% higher in the group exposed to 3 µg PFAA/g egg compared to the control. This difference was significant at the highest dose (p < 0.01) and there was also a significant trend of increased bursa-somatic index with increasing dose (post-test for linear trend, p < 0.01).

### Internal PFAA concentrations

Three males and one female exposed to vehicle (DMSO), three males and two females exposed to 0.5 µg PFAA/g egg, and four females exposed to 3 µg PFAA/g egg were analysed regarding concentrations of the components of the PFAA mixture in liver and plasma. Since we previously found no sex difference in tissue concentration of PFOS and PFOA in exposed chicken embryos (Mattsson *et al*.^32^ and manuscript in preparation) and there was no indication of sex difference in the present study, the results from females and males were combined. PFAA concentrations in each individual are provided in Supplementary Table S1. All control livers had concentrations below the method detection limit (MDL), except for one embryo which had a concentration of PFHxS just above MDL. In control plasma, 72% of the values were above the MDL and the quantifiable concentrations were up to 11% of the corresponding concentration in the group exposed to 0.5 µg/g egg. All MDL values and tissue concentrations of each individual are provided in the online Supplementary Table S1. The total concentration of all studied PFAAs (group mean ± standard deviation) was 4.6 ± 0.7 µg/g at the lower dose and 24 ± 4 µg/g at the higher dose in liver, whereas in plasma it was 3.9 ± 0.9 µg/g at the lower dose and 23 ± 2 µg/g at the higher dose.

**Figure 5:**
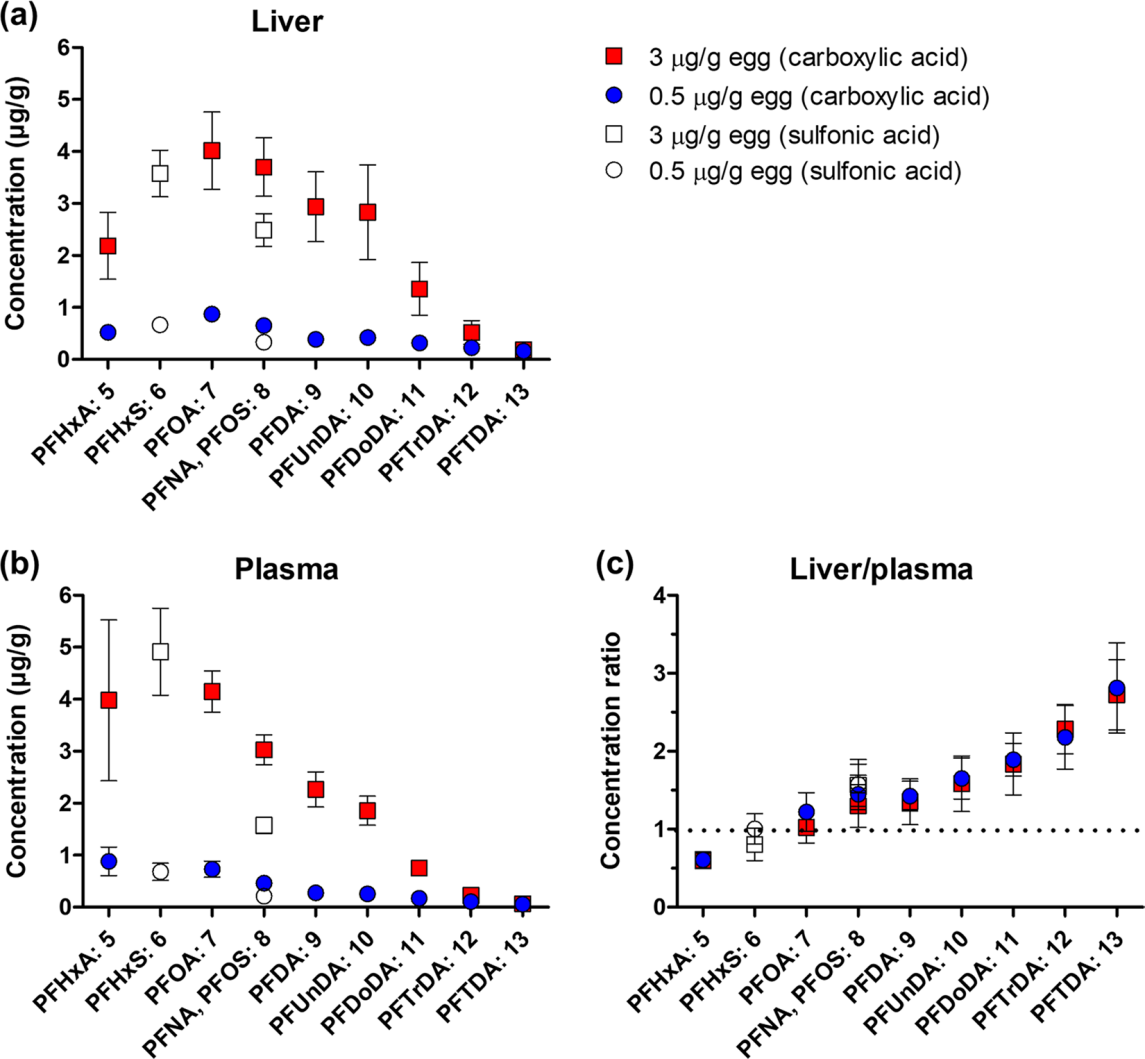
Internal PFAA concentrations in chicken embryos exposed *in ovo* to a mixture of PFAAs at 0.5 and 3 µg/g egg of each substance. (**a)** Concentrations in liver. (**b**) Concentrations in plasma. **(c)** Liver/plasma concentration ratios. There was no indication of sex differences, and females and males are merged in these graphs. N = 4–5 embryos/group. Symbols show group mean ± SD. The numbers on the x-axis indicate the number of perfluorinated carbons in the alkyl chain of each substance. The concentrations shown for PFHxS and PFOS are the sums of branched and linear isomers. In control embryos (not shown in the graphs), the concentrations were below or just above MDL in liver whereas in plasma the concentrations were up to 11% of the concentration found in the low-dose group (see Supplementary Table S1). The dotted line in (**c**) indicates a liver/plasma concentration ratio of one, i.e. values above this line indicate a preferential distribution to liver whereas values below indicate a preferential distribution to plasma. The concentrations in liver and plasma decreased with increasing perfluoroalkyl chain length for long-chain carboxylic acids (linear regression, p < 0.001) and sulfonic acids (Bonferroni’s post-test, p < 0.01) at both injected doses. The liver/plasma concentration ratio increased with increasing chain length for carboxylic acids (p < 0.001) and sulfonic acids (p < 0.01) at both injected doses.

Figure 5 shows the concentration of each compound in liver and plasma as well as the liver/plasma concentration ratio. The substances are ordered on the x-axis according to increasing number of perfluorinated carbons, i.e. perfluoroalkyl chain length. In the sulfonic acids (PFHxS and PFOS), all carbons (n) are perfluorinated (represented by open symbols). The carboxyl group of the carboxylic acids is not fluorinated, and thus the number of perfluorinated carbons is n-1 for these substances. Linear regression analysis showed that concentrations in liver and plasma of long-chain carboxylic acids decreased with increasing chain length at both doses (p < 0.001). PFHxA, which was the only short-chain PFAA included, deviated somewhat from the trend by showing lower mean concentrations in liver than several of the carboxylic acids with longer chains. At the higher dose, liver concentrations of PFHxA were significantly lower than those of PFOA, perfluorononanoic acid (PFNA), perfluorodecanoic acid (PFDA), and perfluoroundecanoic acid (PFUnDA) (p < 0.05). The shorter sulfonic acid (PFHxS) showed lower concentrations than the longer (PFOS) in both liver and plasma (p < 0.001 at 0.5 and <0.01 at 3 µg/g egg). PFOS showed lower concentrations than the carboxylic acid with the same number of perfluorinated carbons, i.e. PFNA, in both liver and plasma at both doses (p < 0.01). The mixture lacked a carboxylic acid with six perfluorinated carbons but the concentrations of the sulfonic acid PFHxS fitted fairly well the pattern of the carboxylic acids.

The six-fold difference between the injected doses (0.5 and 3 µg/g egg) was relatively well mirrored by the internal concentrations in liver and plasma for all substances except for the three with the longest alkyl chains, perfluorododecanoic acid (PFDoDA), perfluorotridecanoic acid (PFTrDA) and perfluorotetradecanoic acid (PFTDA) (Fig. 5a,b and Supplementary Table S1). The plasma concentrations of all compounds except for these three differed significantly between the two exposure groups (p < 0.001). In liver, all but PFTrDA and PFTDA differed significantly in concentration between the two exposure groups (p < 0.001).

Linear regression analysis of liver/plasma concentration ratio showed that this ratio increased with increasing chain length for both carboxylic acids (p < 0.001) and sulfonic acids (p < 0.01) at both doses. PFAAs with eight or more perfluorinated carbons had a liver/plasma concentration ratio larger than one, i.e. they showed a preferential distribution to liver (Fig. 5c). Supplementary Fig. S1 shows the relationships between liver and plasma concentrations of the PFAAs of varying chain lengths. Liver and plasma concentrations in exposed embryos were significantly correlated for all compounds (Pearson’s correlation; p < 0.001).

The perfluoroalkyl sulfonic acids used in this study contained both linear and branched isomers. The PFOS stock solution was analyzed and contained 17% branched isomers. The proportion of branched PFOS isomers in liver and plasma ranged from 14% to 20%; these analytical results are provided in the online Supplementary Table S2. The stock solution of PFHxS was not analyzed, but the proportion of branched isomers in liver and plasma was similar to that of PFOS (12–18%).

## Discussion

### General toxicity

In the present study we explored effects of embryonic exposure from E4 to E18 to a mixture of ten different PFAAs (all long-chain except PFHxA) using the chicken as a model. The PFAA mixture did not affect survival, liver weight or body weight and there were no signs of developmental delay at the day of dissection (E18), suggesting no major effects on growth and general development.

### Thyroid effects

The PFAA mixture disrupted thyroid function, as indicated by significantly decreased serum levels of thyroid hormones, decreased height of the follicular epithelium, and increased mRNA expression of *DIO3* compared to the vehicle control. There were also statistically significant trends toward decreased size of the thyroid follicles and a decreased relative colloid area with increasing dose.

Thyroid hormones are essential for normal development and differentiation of almost every organ and for regulation of growth and basal energy metabolism throughout life across vertebrate species. Because of the critical roles of thyroid hormones during early development in vertebrates, even small changes in their signaling can cause adverse effects that persist or become evident later in life^33^. In precocial birds such as the chicken, thyroid hormones regulate growth, tissue differentiation and maturation during embryonic life as well as post-hatch^34^. Blood concentrations of thyroid hormones increase sharply during the latter half of embryonic life, and the perihatch peak in concentrations (mainly T4)^35^ is required for preparing the chick for hatching and for the transition to life outside the egg^36,37^. In contrast to the situation in mammals, where T3 is much more potent than T4, these hormones have similar physiological potencies in birds^34^.

In the present study, serum concentrations of free thyroid hormones (i.e. fraction not bound to blood carrier proteins) were analyzed on E18. This is three days before anticipated hatching and at a stage of development when the thyroid hormone levels are sharply rising^35,38^. Both fT3 and fT4 were significantly reduced by the highest dose of the PFAA mixture (3 µg/g egg) compared to the vehicle control. The largest effect was found for fT4, for which the concentration decreased by more than 50%, whereas the decrease of fT3 was 23%. There were also significant linear trends of a decrease of fT4 and fT3 with exposure dose across all groups, indicating that there may be some treatment effect also at lower doses.

Considering the importance of the thyroid hormones in the transition of the embryo to post-hatch life, the observed hormonal changes may result in severe consequences that may not be manifested until post-hatch life. For instance, reduced thyroid hormone levels can delay hatching and reduce filial imprinting behavior in the newly hatched chick^37,39^. Interestingly, Pinkas *et al*. (2010) found that embryonic exposure to PFOS and PFOA at 5 µg/g egg (lowest dose tested) reduced hatching success and imprinting behavior in the chicken^40^. The impaired imprinting shown by Pinkas *et al*. (at 5 µg/g egg) and the decreased thyroid hormone levels in the present study (at 3 µg/g egg) suggest that PFAAs can affect imprinting behavior by interfering with the thyroid hormone system.

Concurrent with the observed reduction in circulating thyroid hormone levels in response to the PFAA mixture, there was a significant reduction of the height of the thyroid follicular epithelium (11 and 20% lower than the control mean at 0.5 and 3 μg/g egg). Detection of such effects requires thorough morphometric analysis rather than only visual examination. Using morphometric analysis we also found small but significant negative correlations between PFAA dose and relative colloid area and follicular size.

The observed histological changes of the thyroid gland are indicative of a decreased workload of the gland. T4, and to some extent T3, is produced by the epithelial (follicular) cells of the thyroid follicles. The capacity of the thyroid gland to produce thyroid hormones is limited by size and number of follicular cells and is regulated by thyroid-stimulating hormone (TSH) via the hypothalamic-pituitary-thyroid (HPT) axis, which is well conserved between birds and mammals^34^. Thus, the reduced size/height of the follicular cells in the present study along with the slight reduction of follicular size may have contributed to the reduction of serum thyroid hormone levels. The decrease in relative colloid area suggests slightly reduced hormone storage.

The effect of the PFAA mixture on circulating thyroid hormone levels in our avian embryo model is in line with effects of exposure to single PFAAs in mammalian models. Experiments performed in cynomolgus monkey^41,42^, rat and mouse^43–46^ have shown that exposure to PFOS or PFOA causes reduction in circulating levels of T3 and T4. This usually occurred without a compensatory feedback increase in TSH. Moreover, exposure of pregnant rat dams resulted in reduced maternal serum levels of T4 (free and total) and T3^45^ and in attenuation of the ontogenetic increase in serum total T4 (tT4) and fT4 in their pups the first two weeks of postnatal life^47^. A similar pattern of reduction in T4 was also seen in pregnant mice, although no effect on T4 was found in their pups^45,47^.

The mechanism behind decreased thyroid hormone serum levels by PFAAs has been suggested to involve displacement of the hormones from carrier proteins followed by increased turnover^48^. Weiss *et al*. (2009) found that various PFAAs were able to compete with and displace T4 from the human thyroid hormone transport protein transthyretin (TTR)^49^. Furthermore, Chang *et al*. (2008) demonstrated that PFOS caused a transient increase in serum fT4 in rats followed by an increased turnover that, after equilibrium between fT4 and tT4 was reached, resulted in a reduction in serum tT4^46^. Birds lack the thyroxine-binding globulin (TBG) found in large mammals (but not rodents) and TTR and albumin are the main transport proteins for thyroid hormones in birds^34^. Thus, interaction with TTR is of relevance also for birds and may have contributed to the decrease in thyroid hormone concentrations found in this study. Other mechanisms may also have been involved in the effects on hormone levels; the reduction in fT3 and fT4 in our avian model occurred concurrently with hypotrophy of thyroid follicular cells, suggesting a reduced thyroid hormone synthesis.

It cannot be excluded that the reduction in serum levels of fT4 seen after exposure to the PFAA mixture was influenced by a possible negative bias in the analog competitive immunoassay used for measuring the hormone. Chang *et al*. (2007) showed in a rat study that an observed association between high serum levels of PFOS (49 µg/ml) and reduced fT4 levels using analog methods may be inaccurate because of displacement of fT4 and labelled analog from serum carrier proteins by PFOS^50^. On the contrary, Lopez-Espinosa *et al*. (2012) found no such methodological problems with the analog competitive immunoassay when analyzing fT4 in serum samples from a human population with background PFOS concentrations (median = 9.6 ng/ml) and elevated PFOA concentrations (42 ng/ml)^51^. The authors speculated that the differences in results may be due to a difference in serum PFOS concentrations between the studies and/or species differences in binding capacity of the serum carrier proteins. That the observed reductions in fT3 and fT4 following PFAA exposure in the present study are real, and not due to an analytical inaccuracy, is supported by the concurrent effects on thyroid histology.

### Gene expression

We explored effects of the PFAA mixture on hepatic mRNA expression of genes known to be involved in the thyroid hormone signalling pathway or lipid metabolism (which is partly regulated by thyroid hormones). Among the four analysed genes involved in thyroid signalling, *THRA, TTR*, and *DIO1* remained unaltered whereas *DIO3* was upregulated. *THRA* encodes thyroid hormone receptor alpha (TR-α) which is a nuclear receptor that regulates gene expression upon binding to thyroid hormones. The other nuclear thyroid hormone receptor, TR-β (encoded by *THRB*), is not expressed in the liver in birds^34^ and was therefore not analysed. As discussed above, TTR is an important transport protein for thyroid hormones. The type I and type III deiodinases (encoded by *DIO1* and *DIO3*, respectively) are the main deiodinases active in the liver in birds, whereas type II deiodinase is mainly found in the brain^34^ and was not analysed in the present study. Type III deiodinase deactivates T4 to inactive reverse-T3 (rT3) and T3 to diiodothyronine (T2). *DIO3* was significantly upregulated by the lower PFAA dose but not by the higher dose. The mechanism behind this non-monotonic dose-response relationship is unknown but it has been shown that endocrine disrupters can cause non-monotonic responses via various mechanisms^52^. The performed gene expression analysis provides only an instant picture of the expression on E18 and we do not know if the change in the low-dose group was temporary and if there was a change at earlier stages in the high-dose group. The result is however supported by a previous study by Cassone *et al*. who showed that PFHxS at 0.9 µg/g egg (but not PFHxA) increased the hepatic expression of *DIO3* (and *DIO2*) and decreased the plasma levels of fT4 in chicken embryos^28^. A speculation is that elevated levels of Type III deiodinase in the liver, as indicated by increased *DIO3* expression, contributed to the lowered T3 and T4 levels by enhanced T3 deiodination to T2 and T4 deiodination to rT3.

In a recent study, Jacobsen *et al*. (2018) found that exposure of chicken embryos to PFOS at 0.1 and 1 µg/g egg from E4 to E19 caused broad downregulation of genes involved in lipid metabolism in the liver^53^. Two of the most consistently downregulated genes in their study were *ACAA2* and *FABP5*. *ACAA2* encodes acetyl-CoA acyltransferase 2, an enzyme involved in beta-oxidation of fatty acids^54^. *FABP5* encodes fatty acid-binding protein 5, also called epidermal fatty acid-binding protein (E-FABP). FABPs are intracellular lipid chaperones involved in transport of lipid to different compartments of the cell that may play important roles in lipid signaling and metabolic regulation^55^. In contrast to the aforementioned study, we found no effect on *ACAA2* and *FABP5* expression by the PFAA mixture. Notably, the expression of *ACAA2* was approximately 1.8-fold higher in males than in females in the present study, highlighting the importance of taking the sex of the studied individual into account in effect studies. The highest dose of the PFAA mixture induced mRNA expression of *LBFABP*; this transcript encodes liver basic fatty acid-binding protein, which is found in the liver of avian and other non-mammalian species^56^. Other genes involved in lipid and fatty acid homeostasis that were not altered by treatment in the present study were *LXR-A* and *ACOX1*.

### Bursa of fabricius

The bursa-somatic index was significantly lower in female than in male embryos in the control group and in the low-dose group. This sex difference corroborates our findings in a previous study where we found that the diameter of the bursa was significantly larger in male than in female chicken embryos (unpublished results). The PFAA mixture seemed to abolish this sex difference at the highest dose by causing increased bursa-somatic index in females to the male level. There were, however, no apparent sex differences or treatment effects found at the histological level. The bursa of Fabricius is an important immune organ and the primary site of B-lymphocyte development in birds and a proper function of the bursa is therefore critical for an adequate antibody response to an infection^57^. A possible effect of PFAAs on the function of the bursa is in line with results from a study by Peden-Adams *et al*. (2009), showing that *in ovo* exposure of chicken embryos to PFOS caused dramatic effects on both innate and adaptive immune functions^29^. Moreover, O’Brien *et al*. (2013) found that acute phase genes were downregulated in chicken liver by PFUnDA indicating impaired acute phase immune response^58^. These effects warrant further studies to elucidate potential effects of other PFAAs on the bursa of Fabricius and on B-lymphocyte development and function in birds.

PFOS and PFOA have also been implicated in impairment of immune functions in mammalian experimental studies as well as in human epidemiological studies, and the humoral immune response seems to be a particularly sensitive target^59^. The European Food Safety Authority (EFSA) recently concluded that the most critical and consistent effects of PFOS (and possibly also PFOA) in humans is a decrease in antibody response following vaccination in children^60^. It was estimated that a considerable proportion of the EU citizens are at risk of impaired immune function because of PFOS exposure.

### Internal concentrations

The internal concentration of each component of the PFAA mixture on the day of dissection was determined in plasma and liver. The carboxylic acids showed decreasing concentrations in both tissues with increasing carbon chain length from PFOA to PFTDA (seven and thirteen perfluorinated carbons, respectively). A similar pattern has also been found regarding maternal transfer of PFAAs via placenta and milk as studied in infants and their mothers; transfer decreased with increasing chain length from around seven perfluorinated carbons^24^. By contrast, it has been shown that in some (but not all) wild bird species maternal to egg as well as egg to embryo transfer increase with increasing perfluoroalkyl chain length^16,20,21^. The accumulation pattern in our study also differs from that one would expect from bioaccumulation data on PFAAs in field and laboratory studies of other organisms. Within the homologous series of carboxylic acids and sulfonic acids, bioconcentration factors (BCFs) and biomagnification factors (BMFs) generally increase with increasing perfluoroalkyl chain length up to a chain length of 10–13 perfluorinated carbons, after which they level off or even decrease^4,5,7,61,62^. Whether the discrepancy between the accumulation pattern in our avian model and the bioaccumulation patterns in many other organisms reflects differences in uptake and/or excretion in relation to perfluoroalkyl chain length demands further studies. Possibly, the relatively low concentrations of PFAAs with longer chains in the embryos in our study could be due to limited passage of these substances through the inner shell membrane after air sac injection. Injection of PFOS and PFOA via yolk (mimics maternal transfer in birds) or via air sac results in approximately the same internal concentrations (unpublished results), suggesting that the uptake of these PFAAs does not differ between yolk and air sac.

Another pattern that was evident from our results was a preferential distribution to liver compared to plasma with increasing perfluoroalkyl chain length. This result agrees well with the distribution pattern of carboxylic acids with up to ten perfluorinated carbons between liver and plasma in herring gulls^20^.

The PFOS exposure solution contained approximately 17% branched isomers. In contrast to the situation in a fresh-water ecosystem where branched isomers have been shown to be depleted in biota^8^, the fraction of branched isomers in the liver and plasma of the chicken embryos did not change from that of the exposure solution. Depletion of branched isomers in biota is attributed to their higher water solubility and faster excretion^63^.

### Concluding remarks

We have shown that developmental exposure to a mixture of PFAAs (mainly long-chain) affected endpoints related to the thyroid hormone and immune systems in an avian model. These effects are in line with thyroid hormone disruption and immunotoxicity by e.g. PFOA and PFOS found in rodents and humans^59,64^. The lowest dose tested, 0.5 μg/g egg, caused reduced thyroid follicular epithelial height and altered mRNA expression of *DIO3*. Effects on thyroid hormone serum levels at the higher dose corroborates a functional effect on the thyroid hormone system. PFAA concentrations in wild birds are usually in the lower ng/g range, but in highly exposed populations low µg/g concentrations are found in eggs and in tissues of embryos and adult birds^15,16,65,66^. Highly exposed bird populations may therefore be at risk for thyroid hormone disruption due to developmental exposure. We do not know if the observed effects were mainly due to one component of the mixture or if it resulted from additive effects of multiple components. Nevertheless, our results highlight the vulnerability of the developing thyroid and immune systems in birds and call for further studies of single PFAAs as well as other emerging PFASs.

## Methods

### Chemical mixture

Chicken embryos were exposed to a mixture of ten different PFAAs (eight carboxylic acids and two sulfonic acids) at equal concentrations in dimethyl sulfoxide (DMSO; ≥95% purity; Sigma-Aldrich, St. Louis, MO, USA). The studied doses (0.5 and 3 µg/g egg of each compound) were based on levels in wild birds (eggs and tissues) and effect doses in chicken embryos after exposure to single PFAAs (see introduction and discussion). We chose to inject the components at an equal dose since in organisms their relative concentrations change over time and differ depending on species, population, tissue and life-stage. The PFAAs were perfluorohexanesulfonic acid potassium salt (PFHxS; CAS: 3871-99-6; ≥98% purity; Sigma-Aldrich), perfluorohexanoic acid (PFHxA; CAS: 307-24-4; ≥97% purity; ABCR GmbH & Co, Karlsruhe, Germany), perfluorooctanesulfonic acid (PFOS; CAS: 1763-23-1; ≥97% purity, ABCR), perfluorooctanoic acid (PFOA; CAS: 335-67-1; ≥96% purity; Sigma-Aldrich), perfluorononanoic acid (PFNA; CAS: 375-95-1; ≥97% purity; Sigma-Aldrich), perfluorodecanoic acid (PFDA; CAS: 335-76-2; ≥98% purity; ABCR), perfluoroundecanoic acid (PFUnDA; CAS: 2058-94-8; ≥95% purity; Sigma-Aldrich), perfluorododecanoic acid (PFDoDA; CAS: 307-55-1; ≥96% purity; ABCR), perfluorotridecanoic acid (PFTrDA; CAS: 72629-94-8; ≥97% purity; Sigma-Aldrich), and perfluorotetradecanoic acid (PFTeDA; CAS: 376-06-7; ≥97% purity; ABCR). All these substances are classified as long-chain PFAAs, except for PFHxA, which is defined as a short-chain PFAA^3^.

### *In ovo* exposure

The experimental procedures were approved by the Uppsala Ethical Committee for Research on Animals (permit number C 90/15) and carried out in accordance with guidelines by the Swedish Board of Agriculture. The facilities used for egg incubation and dissection were approved by the Swedish Board of Agriculture (permit number 5.2.18-11059).

Chicken embryos *(Gallus gallus domesticus)* were exposed to the PFAA mixture by *in ovo* injection. By injecting on embryonic day 4 (E4) and assessing effects on E18 (three days before anticipated hatching), the exposure covers most of the embryonic development including the functional development of bursa of Fabricius and thyroid hormone system^34,57^. By E4 it is easy to determine whether eggs are fertilized or not.

Chicken eggs were purchased from Ova Production AB, Vittinge, Sweden. To limit the number of embryos to be dissected in one day, two replicate experiments were carried out on two consecutive days. Eggs were randomly allocated to the two replicates, and were incubated horizontally at 37.5 °C and 60% relative humidity. The eggs were turned automatically every six hours. The day the eggs were placed in the incubator was defined as embryonic day zero (E0).

On E4, the eggs were candled. Unfertilized eggs, eggs with cracks in the shell and eggs containing very small or dead embryos were discarded. A normally developed embryo appears at candling as a dark red spot with blood vessels radiating out from it, whereas a dead embryo is coagulated (brown spot) or is indicated by a ring or streaks of blood in the egg (Supplementary Fig. S2). The rate of eggs with normally developed embryos was 77/92 (84%). The embryonated eggs were randomly allocated to three different groups and exposed via air sac injection to either DMSO (vehicle control; N = 11 + 10), PFAA mixture at 30.7 µg/egg (N = 11 + 10) or PFAA mixture at 184 µg of each compound/egg (N = 11 + 9). The weight of 46 randomly chosen eggs on E0 was 63 ± 4 g (mean and standard deviation) and the PFAA doses therefore correspond to approximately 0.5 and 3 µg/g egg. Air sac injection was performed as follows: the blunt end of each egg was wiped with ethanol and a small hole was drilled through the shell above the air sac (shown in Supplementary Fig. S2). Then, 20 μl injection solution was deposited onto the inner shell membrane in the air sac using a Hamilton syringe and the hole was sealed with melted paraffin wax. Following injection, the egg was immediately placed horizontally. We have previously noted that DMSO causes increased mortality after air sac exposure at early stages if the egg remains in a vertical position even for only a few minutes. The eggs were then labeled with individual identity numbers and returned to random locations in the incubator.

### Embryo dissection and sampling

Embryos were dissected and sampled on E18, approximately three days before anticipated hatching. Dissection was done in randomized order and blinded to treatment. Mortality rates were noted and living embryos were euthanized by decapitation and examined for gross external and internal morphological changes. Weights of the body, liver, spleen, bursa of Fabricius, and left thyroid gland were recorded. The relative organ weights (%) were calculated as 100 × organ weight/body weight. Bursa, left thyroid gland and a piece of liver were taken and processed for histology as described below. Another sample of the liver was placed in RNA-later (Sigma-Aldrich) and stored at −20 °C until further analysis of gene expression. Two or three randomly chosen embryos from each group and replicate day were sampled for analysis of PFAAs in blood plasma and liver. Blood was collected from the allantoic/umbilical artery before decapitation as described previously^32^ and the blood cells were then separated from the plasma by centrifugation (1300 g; 10 min; 4 °C). Plasma and liver samples were stored at −20 °C until extraction and analysis of PFAAs. The sex of the embryos was determined by the presence of a pair of testes or an ovary and left Müllerian duct.

### Tissue preparation for histology

Liver pieces, bursas and thyroid glands were fixed in phosphate-buffered formalin (4% formaldehyde in 0.1 M phosphate buffer, pH 7.4; v/v), and thereafter dehydrated by treatment with ethanol in a series of increasing concentration (70%, 95%, and absolute ethanol; v/v) followed by soaking in xylene. The dehydrated tissue was embedded in Technovit 7100 (Heraeus Kulzer, Hanau, Germany) and sectioned (2 µm). Bursa and thyroid gland were sectioned along their longitudinal axis. Sections were taken at one level of the liver sample and at two central levels in the bursa (separated by ~300 µm) and thyroid gland (separated by 100–150 µm). Three sections from each level were mounted on Superfrost glass slides and stained with hematoxylin and eosin. One of these three sections was analysed. Sections from bursa and thyroid gland were photographed at tenfold magnification using a Leica LEITZ DMRXE microscope with a DFC550 camera and the software Leica Application Suite 4.2.0 (Leica Microsystems, Wetzlar, Germany). To cover the entire section, approximately four overlapping pictures were taken of the thyroid gland section and 20–25 pictures were taken of the bursa. The pictures were merged using the open source software GNU Image Manipulation Program (GIMP) 2.8. The image analyses were performed using the open source software ImageJ.

### Liver histology

Livers from 17 control embryos and 16 embryos from each of the two PFAA groups were subjected to a brief histological examination by microscopy (Leica LEITZ DMRXE) at a single level. Both females and males were included. The livers were examined for pathological changes, including fatty change, disorganisation of tissue structure, necrosis/apoptosis, and invasion by leucocytes.

### Bursa histology

Histological sections of the bursa of Fabricius were analyzed regarding follicular size and follicle density. The average of the values from the two tissue levels was calculated. The bursa follicular size was defined as the average area (in mm^2^) of individual follicles in the histological sections. Only follicles that were located at the crossing points of an applied grid (area of each square of the grid = 0.2 mm^2^) were analyzed, i.e. 20–30 follicles per section. The outline of the follicle was traced with a tracing tool and the encircled area was then measured. The bursa follicle density was defined as the number of follicles per mm^2^ of plical tissue. A grid was applied to the image and the tissue within four pre-selected squares of 0.5 mm^2^ was analyzed; follicles located in the square were counted and the number was divided by the measured plical area. There were 40–100 follicles in total in all four squares.

### Thyroid gland histology

Histological sections of the thyroid gland were analyzed regarding relative colloid area, follicular size and follicular epithelial height. The average of the values from the two tissue levels was calculated. Relative colloid area was defined as the percentage of the entire thyroid gland section that consisted of follicular colloid and was determined using a pixel intensity threshold for selection and analysis. The thyroid follicular size was defined as the average area (mm^2^) of the individual follicles, including the follicular epithelium, in the histological section. Selection of follicles to be analyzed and the method for analyzing individual follicle area was as described for the bursa, except that the grid square area was set to 0.025 mm^2^. The follicular epithelial height was measured using the line tool at four locations around the follicle: 0°, 90°, 180°, and 270°. There were 70–100 follicles analyzed regarding individual follicular size and epithelial height in each thyroid gland section.

### Thyroid hormone concentrations

In a second experiment, chicken embryos were exposed *in ovo* to the PFAA mixture (0.5 and 3 µg/g egg; same doses as in the first experiment) in order to explore potential effects on circulating levels of thyroid hormones. Embryos were exposed and incubated until E18 as described for the first experiment. Both experiments were performed in the winter (December and March, respectively). In order to limit variations in hormone concentrations related to seasonal or circadian rhythms (sampling time of the day) the eggs were incubated in darkness. Blood was collected from the allantoic artery as described previously^32^ and was allowed to clot at room temperature for 20–60 min. The clotted blood was stored on ice for up to four hours. The clot was then separated from the serum by centrifugation at 1,300 g in a refrigerated centrifuge (4 °C) for 10 min, and the serum was transferred to a new tube, and stored at −20 °C until analysis.

Concentrations of free T4 and T3 were analyzed at an accredited clinical diagnostic laboratory (Department of Clinical Chemistry and Pharmacology at Uppsala University Hospital, Sweden) using an electrochemiluminescence immunoassay (ECLIA) on a Cobas e602 instrument (Roche Diagnostics, Indianapolis, IN, USA). The coefficients of variation (CV) for repeatability in the analyzed ranges were 3% for T3 and 4% for T4.

Some serum samples were pooled in order to obtain sufficient volume (150 µl) for analysis. The results from the pools did not differ from the individual samples and they were therefore treated the same as the individual samples in the statistical analyses. In total, 60 samples including eight pools (at least one pool for each treatment group and sex) were analyzed.

### Gene expression analysis

The mRNA expression in liver was analyzed using real-time quantitative PCR (qPCR) as described in Supplementary Methods available online. The analyzed genes were *THRA*, *DIO1*, *DIO3*, *TTR*, *LXR-A*, *ACAA2*, *FABP5, LBFABP*, and *ACOX1*. The expression of each of these genes in a sample was normalized to the expression of *ACTB* (beta-actin) and *EEF1A1* (eukaryotic translation elongation factor 1 alpha 1). *ACTB* and *EEF1A1* showed little variation between samples and were not affected by treatment and were therefore regarded as appropriate reference genes. Each sample was also normalized to the mean expression level in the control group. GenBank mRNA accession number, primer sequences, product sizes and references are found in Supplementary Table S4.

### Internal PFAA concentrations

The concentrations of all ten components of the PFAA mixture were determined in plasma and liver collected from 4–5 individuals from each exposure group at termination of the experiment on E18. Plasma and liver samples were thawed and liver was homogenized using a cryo homogenizer (Covaris model CP02, MA, USA). Subsamples of 0.15 g liver and 0.05 g plasma were taken and ^13^C- or ^18^O-labelled standards of all target PFAAs (10 ng, Wellington Laboratories, Guelph, Canada), except for PFTrDA which was not available as labelled standard, were added. The samples were extracted using acetonitrile, vortex mixing, and ultrasonication. Extracts from liver samples were subjected to clean-up using dispersive carbon (SupelClean ENVI-Carb, Sigma Aldrich). Extracts were prepared for analysis by addition of milli-Q water and a new set of labelled standards (2 ng) and injected on an Acquity UPLC-TQ-S MS/MS instrument (Waters Corporation, Milford, USA). Procedural blanks, i.e. milli-Q water added instead of sample, were extracted with the samples and the method detection limit was calculated based on the average signal in the blanks and adding three standard deviations, or based on the lowest concentration in an external standard calibration curve. Concentrations were calculated using isotope dilution with single point standards prepared for each batch; these were controlled with a nine-point external calibration curve prepared every three months covering the concentration range of the sample extracts (from 0.01 to 50 ng/mL). The solvent to milli-Q water ratio of the standard solutions were the same as that of the samples. Recoveries of labelled standards were 70–100% for plasma samples and 30–90% for liver samples. For PFHxS and PFOS, both linear and branched isomers were quantified using their respective linear isomer as standard. The method detection limit (MDL) varied between 0.0017 and 0.052 µg/g in liver and between 0.0003 and 0.001 µg/g in plasma, depending on substance. The procedural blanks were below MDL for all analysed substances.

### Statistics

Relationships between perfluoroalkyl chain length of the PFAAs and their internal concentrations were analysed using linear regression. Pearson’s correlation was determined for internal concentrations of individual PFAAs in liver vs. plasma. Differences in concentrations of individual PFAAs were analysed using two-way analysis of variance (ANOVA) with treatment dose and compound as independent variables, followed by Bonferroni’s multiple comparisons post-hoc test to adjust for multiple comparisons. Differences related to treatment effects and/or sex were first analysed with two-way ANOVA with treatment and sex as independent variables. The two-way ANOVA was followed by Bonferroni’s multiple comparisons post-hoc test. In cases where sex differences or interaction between treatment and sex were found, females and males were analysed separately and otherwise they were combined. Differences between the treatment groups and the control were tested using a one-way ANOVA followed by Dunnett’s multiple comparisons post-hoc test and post-test for linear trend. If a sex difference or interaction effect was indicated, males and females were analysed separately. Data that deviated from normal distribution or showed differences in variance between groups was log-transformed prior to analysis. If transformation did not result in normal distribution and homoscedasticity, data was analysed using the non-parametric Kruskal-Wallis test followed by Dunn’s multiple comparisons post-hoc test. Differences were considered statistically significant if p < 0.05. Statistical analyses were performed using GraphPad Prism5 (GraphPad Software Inc, San Diego, CA, USA).

## Supplementary information

Supplementary Information

Supplementary Information 2

Supplementary Information 3

## Data Availability

The datasets generated and analysed in the current study are available from the corresponding author on reasonable request.
