# Supplementary material for: Developmental exposure to a mixture of perfluoroalkyl acids (PFAAs) affects the thyroid hormone system and the bursa of Fabricius in the chicken
Source: Sci Rep. 2019 Dec 24;9:19808. doi: 10.1038/s41598-019-56200-9 (PMC6930258; doi:10.1038/s41598-019-56200-9)
Supplement: Supplementary file 1 — Supplementary Information [file 41598_2019_56200_MOESM1_ESM.docx]

# Supplementary methods

# Developmental exposure to a mixture of perfluoroalkyl acids (PFAAs) affects the thyroid hormone system and the bursa of Fabricius in the chicken

Anna Mattsson^1*^, Sofia Sjöberg^1^, Anna Kärrman^2^, and Björn Brunström^1^

^1^Department of Environmental Toxicology, Uppsala University, Uppsala, Sweden.
^2^ School of Science and Technology, Örebro University, Örebro, Sweden.

^*^Corresponding author: Anna.Mattsson@ebc.uu.se

## Gene expression analysis

Gene expression in liver was analyzed using real-time quantitative PCR (qPCR). The analyzed genes were *THRA* (thyroid hormone receptor, alpha), *DIO1* (iodothyronine deiodinase 1), *DIO3* (iodothyronine deiodinase 3), *TTR* (transthyretin, transcript variants 1 and 2), *LXR-A* (liver X receptor alpha), *ACAA2* (acetyl-CoA acyltransferase 2), *FABP5* (fatty acid binding protein 5), *LBFABP* (liver basic fatty acid binding protein), and *ACOX1* (acyl-CoA oxidase 1). *ACTB* (beta-actin) and *EEF1A1* (eukaryotic translation elongation factor 1 alpha 1) were used as internal control genes. *ACTB* was analyzed using two different primer pairs, giving similar results. Gene-specific primers were designed using the NCBI primer blast tool, which uses the primer3 transcript, or were obtained from other publications. The primers were synthesized by Sigma-Aldrich. GenBank mRNA accession number, primer sequences, product sizes and references are found in Supplementary Table S4 online.

Liver pieces of 10-20 mg were homogenized using a Bullet Blender Storm 24 (Next Advance, Inc., New York, USA) at speed 8 for 2 min in 300 µl lysis solution and a volume of zirconium oxide bullets (0.5 mm) similar to sample volume. After homogenization, additional 500 µl lysis solution was added. Total RNA was isolated using the Aurum total RNA mini kit (Bio-Rad Laboratories Inc. Hercules, CA, USA) with the spin protocol according to manufacturer’s instructions. DNase treatment was included in the protocol to get rid of possible remaining DNA. The RNA was eluted in 80 µl elution buffer. Concentration and purity of RNA were analyzed using a Nanodrop 2000c spectrophotometer (Thermo Scientific NanoDrop products, Wilmington, DE, USA). Integrity of RNA was checked on 1.2 % agarose gel containing GelRed nucelic acid gel stain (Biotium, Fremont, CA, USA) after denaturation in formamide at 70°C for one min. All RNA samples had intact and clear 28S and 18S bands, indicating good integrity of the RNA. Exposure groups and sexes were equally represented at each RNA isolation occasion. The RNA was stored at -80 °C.

cDNA was synthesized by reverse transcription of 450 ng RNA using the iScript cDNA synthesis kit (Bio-Rad Laboratories Inc. Hercules, CA, USA) according to the manufacturer’s protocol. The samples were reverse transcribed in duplicates in a randomized order, and then diluted 25 times in nuclease-free water. After verification by real-time qPCR of the reference gene *ACTB* that the duplicates were similar, they were pooled. This was a quality check included to ensure that the cDNA synthesis was not a major source of variation.

The qPCR was performed using the Rotor-Gene 6000 DNA amplification system (Qiagen, Hilden, Germany). The 20-µl qPCR reaction mixture consisted of cDNA transcribed from 7.2 ng RNA, 0.2 µM forward and reverse primers, and iQ SYBR green supermix at concentration specified in the protocol (Bio-Rad laboratories Inc. Hercules, CA, USA).

The qPCR-program included an enzyme activation step for 3 min at 95 °C and 35 cycles of 15 s at 95 °C and 45 s at primer-specific annealing temperature (59-62 °C). The program ended with a melt curve analysis from 55°C to 95°C to confirm the presence of a single amplicon. Annealing temperature was optimized for each primer pair to obtain specificity and a high reaction efficiency (~2).

All samples were analyzed in the same qPCR run and two runs were done per gene. Controls without template (NTC) and controls without reverse transcriptase (RTC) were included in all runs to ensure that there was no contamination of PCR products in the reagents or amplification of genomic DNA in the samples, respectively.

Relative gene expression was calculated applying the E^-ΔΔCt^ method^1^. The efficiency (E) of each primer pair was determined using a dilution series of cDNA. The mean value of *ACTB* and *EF1A1* in a sample was used for normalizing the transcription levels of the analyzed genes. The control group was used as a calibrator, i.e. the expression level in each sample was normalized to the mean expression level in the control group.

## Reference

1 Livak, K. J. & Schmittgen, T. D. Analysis of Relative Gene Expression Data Using Real-Time Quantitative PCR and the 2−ΔΔCT Method. *Methods* **25**, 402-408, doi:<https://doi.org/10.1006/meth.2001.1262> (2001).
