# Supplementary material for: Developmental exposure to a mixture of perfluoroalkyl acids (PFAAs) affects the thyroid hormone system and the bursa of Fabricius in the chicken
Source: Sci Rep. 2019 Dec 24;9:19808. doi: 10.1038/s41598-019-56200-9 (PMC6930258; doi:10.1038/s41598-019-56200-9)
Supplement: Supplementary file 2 — Supplementary Information 2 [file 41598_2019_56200_MOESM2_ESM.pdf]

Supplementary Figures

Developmental exposure to a mixture of perfluoroalkyl acids (PFAAs) affects the thyroid hormone system and the bursa of Fabricius in the chicken

Anna Mattsson[1*], Sofia Sjöberg[1], Anna Kärrman[2], and Björn Brunström[1]

[1]Department of Environmental Toxicology, Uppsala University, Uppsala, Sweden.
[2] School of Science and Technology, Örebro University, Örebro, Sweden.

[*]Corresponding author: Anna.Mattsson@ebc.uu.se

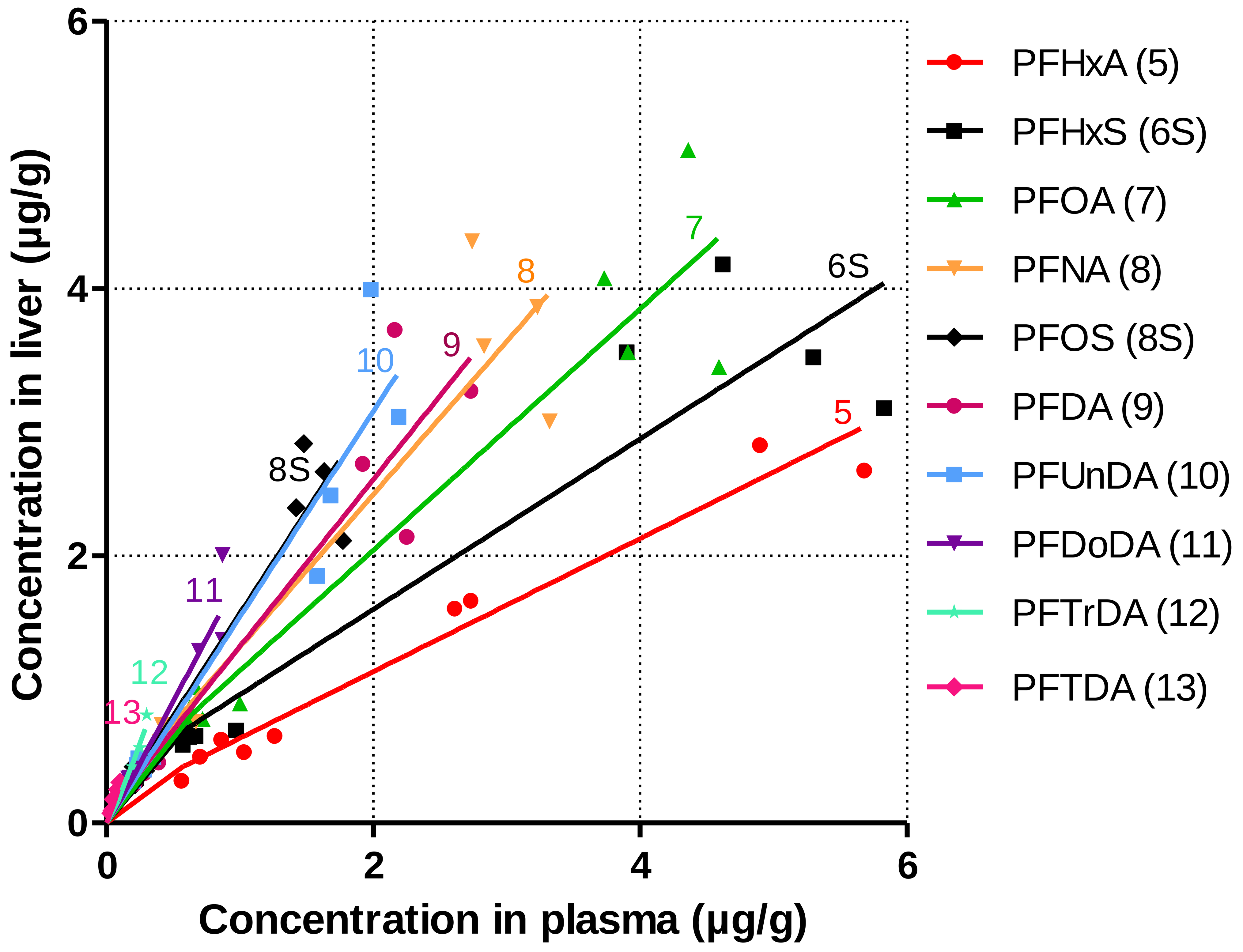

Figure S1. Relationships between liver and plasma concentrations of PFAAs in chicken embryos. PFAA concentrations were measured in embryos of both sexes exposed *in ovo* to the vehicle control (DMSO), or a mixture of PFAAs at 0.5 or 3 µg/g egg of each substance. Four or five embryos from each group were analyzed. The relationship for each substance is shown by a symbol and a fitted line. The numbers indicate the number of perfluorinated carbons in the alkyl chain of each substance and the two sulfonic acids PFHxS and PFOS are indicated with an S. Liver and plasma concentrations in exposed embryos correlated significantly for all compounds (Pearson's correlation; $p<0.001$). The substances with the longer alkyl chains show a preferential distribution to liver whereas those with shorter chains show a preferential distribution to plasma. PFOS shows a higher liver/plasma ratio than the corresponding carboxylic acid with the same perfluoroalkyl chain length (PFNA).

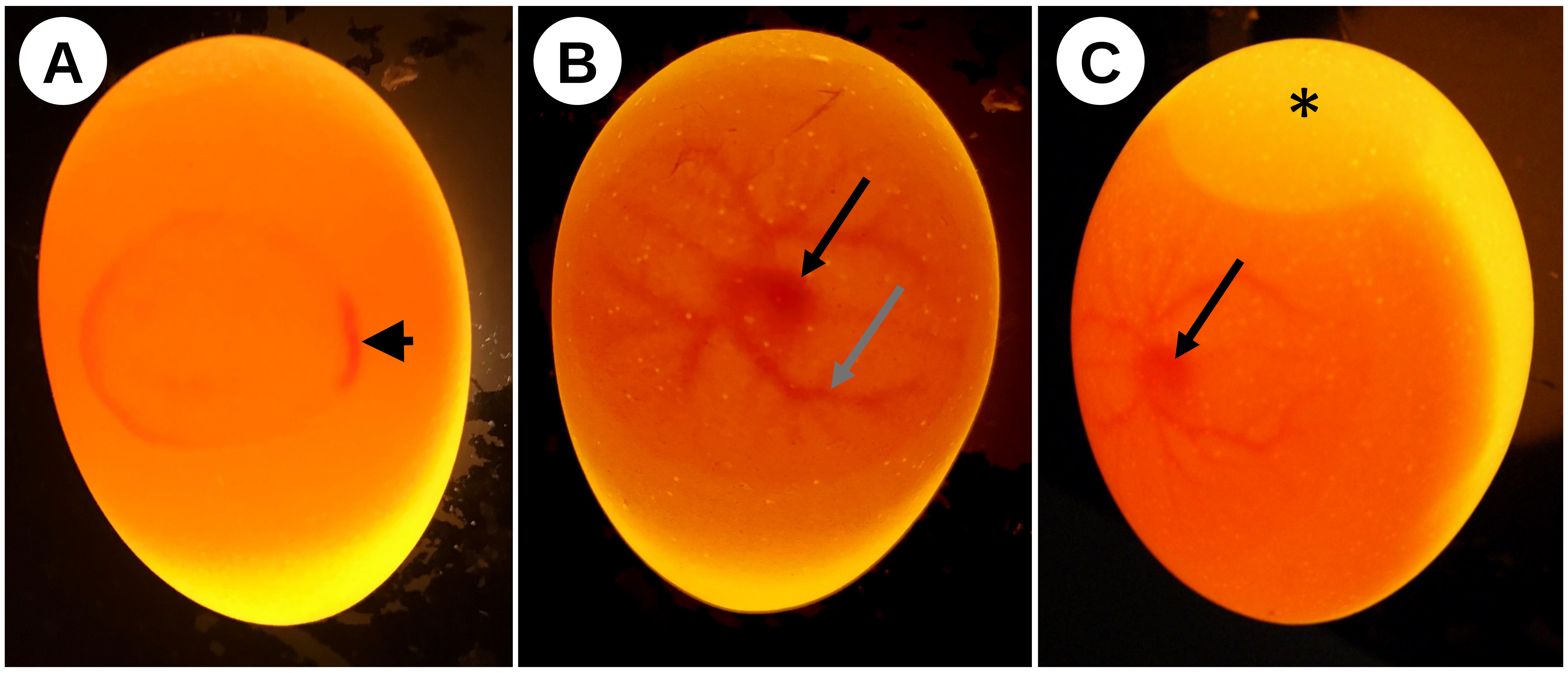

Figure S2. Chicken eggs candled on day four of incubation (E4). A) Egg with a ring of blood (arrowhead), showing that the embryo has died. B) Egg with a viable embryo (black arrow) surrounded by branching vitelline blood vessels (grey arrow). C) The egg is slightly tilted to show the air sack into which the PFAA mixture or vehicle were injected. The black arrow points at the embryo proper and the asterix (*) indicate the air sack.
Photo: Mimmi Wänn and Anna Mentor.
